# On the roles of intrinsically disordered proteins and regions in cell communication and signaling

**DOI:** 10.1186/s12964-021-00774-3

**Published:** 2021-08-30

**Authors:** Sarah E. Bondos, A. Keith Dunker, Vladimir N. Uversky

**Affiliations:** 1 Department of Molecular and Cellular Medicine, Texas A&M Health Science Center, College Station, TX 77843 USA; 2 Center for Computational Biology and Bioinformatics, Department of Biochemistry and Molecular Biology, Indiana University School of Medicine, Indianapolis, IN 46202 USA; 3 Department of Molecular Medicine and USF Health Byrd Alzheimer’s Research Institute, Morsani College of Medicine, University of South Florida, Tampa, FL USA; 4 Institute for Biological Instrumentation of the Russian Academy of Sciences, Federal Research Center “Pushchino Scientific Center for Biological Research of the Russian Academy of Sciences”, Pushchino, Russia

**Keywords:** Amino acid sequence, Protein structure, Disorder prediction, Intrinsically disordered proteins

## Abstract

For proteins, the sequence → structure → function paradigm applies primarily to enzymes, transmembrane proteins, and signaling domains. This paradigm is not universal, but rather, in addition to structured proteins, intrinsically disordered proteins and regions (IDPs and IDRs) also carry out crucial biological functions. For these proteins, the sequence → IDP/IDR ensemble → function paradigm applies primarily to signaling and regulatory proteins and regions. Often, in order to carry out function, IDPs or IDRs cooperatively interact, either intra- or inter-molecularly, with structured proteins or other IDPs or intermolecularly with nucleic acids. In this IDP/IDR thematic collection published in *Cell Communication and Signaling*, thirteen articles are presented that describe IDP/IDR signaling molecules from a variety of organisms from humans to fruit flies and tardigrades (“water bears”) and that describe how these proteins and regions contribute to the function and regulation of cell signaling. Collectively, these papers exhibit the diverse roles of disorder in responding to a wide range of signals as to orchestrate an array of organismal processes. They also show that disorder contributes to signaling in a broad spectrum of species, ranging from micro-organisms to plants and animals.

## Introduction

Intrinsically disordered proteins and regions (IDPs and IDRs) lack stable tertiary structure yet carry out a diverse array of biological functions [1–4]. Probably the first development of this concept was made in 1940 by Pauling [5]. Several experimentally characterized IDPs were reported in the 1950s and 1960s (reviewed in [3, 6]). Interestingly, in 1966, Jirgensens [7] developed a database that partitioned proteins according to their secondary structures as estimated by optical rotatory dispersion. This database included a few unstructured proteins in a disordered category. For some of these proteins, the relative lack of helix and sheet was supplemented with intrinsic viscosity measurements indicating a very elongated shape and by noticing a high net charge, which would reduce foldability.

On the structured protein side, Linderstrøm-Lang [8] used differential rates of protease digestion of variously sized protein fragments and whole proteins to suggest that proteins are organized by a primary, secondary, and tertiary structural hierarchy. Following the first determination of the 3D structure of a protein, myoglobin [9], Linderstrøm-Lang and Schellman [10] mapped the structural features of this protein to the indicated hierarchy. This primary, secondary, tertiary (with the later addition of quaterinary) hierarchy now introduces protein structure in essentially every biochemical textbook. Linderstrøm-Lang and Schellman [10] also discussed disordered proteins as exceptions to this hierarchy.

From the 1970’s to the 1990s, an avalanche of protein structures was determined by X-ray crystallography and collected in the Protein Data Bank (PDB) [11, 12], leading to the (mistaken) view that sequence → structure → function paradigm is likely universally true as an explanation for all protein functions. The early work on IDPs and IDRs was largely forgotten. What is not generally appreciated, however, is that the accumulation of this same set of structured proteins also led to an avalanche of IDRs, which are identified as regions of missing electron density among the structured proteins. Several of these IDRs exhibited interesting and crucial biological functions (reviewed in [3, 6]). Indeed, in a datamining investigation of about 100 such IDRs, about 85 were found to have one or more of 28 different functions [13].

Another important source of IDPs and IDRs has been the Structural Genomics Initiative [14]. IDPs and IDRs occur much more often than originally anticipated [15, 16]. Indeed, fully structured proteins are not common; only about 7% of a non-redundant set of structured proteins spanning the PDB are fully structured without any disordered residues [17]. Roughly 10% of proteins in the PDB contain disordered regions longer than 30 amino acids, and an additional 40% of PDB proteins contain disordered regions between 10 and 29 residues long [17].

**Fig. 1:**
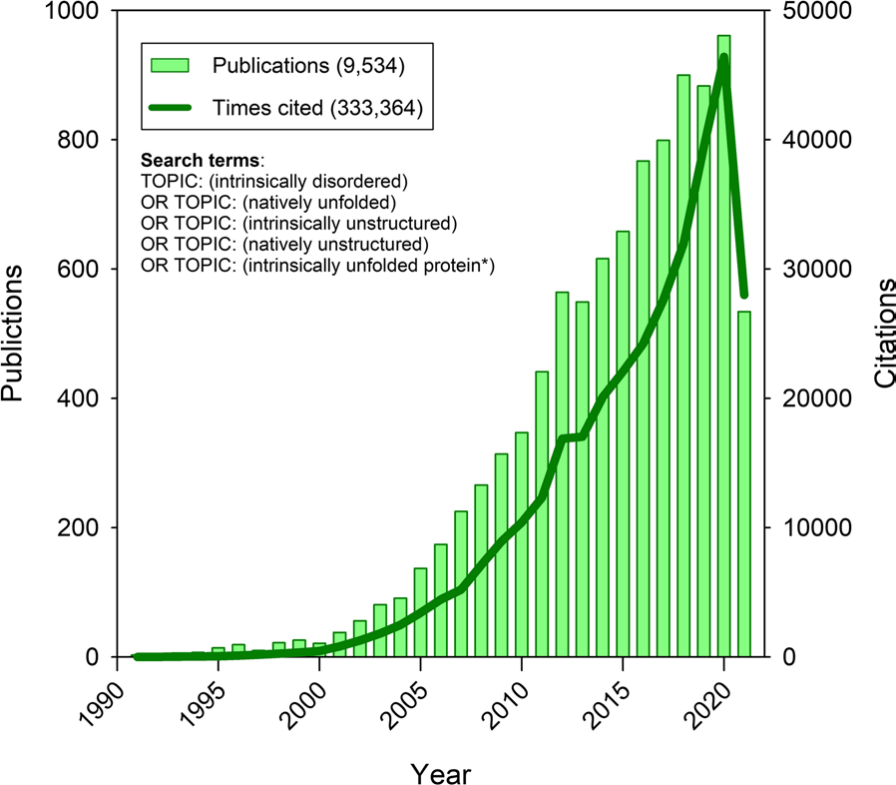
The time-courses of the increase in the number of publications dealing with intrinsic disorder (green bars) and the number of papers citing those publications (green line). Data for these plot were retrieved from Web of Science on August 01, 2021 using the following search criteria: TOPIC: (intrinsically disordered) OR TOPIC: (natively unfolded) OR TOPIC: (intrinsically unstructured) OR TOPIC: (natively unstructured) OR TOPIC: (intrinsically unfolded protein*)

Studies on IDPs and IDRs have been moving towards the mainstream of protein science research in from the mid-1990s to the present. In our view [6], increased use of NMR for protein structure analysis and the application of bioinformatics approaches to better understand IDPs and IDRs have been largely responsible for this movement. The rapid growth since the mid-1990s in the number of publications on IDPs and IDRs shown previously [18] is continuing to the present (see Fig. 1).

The manually curated Database of Disordered Proteins (https://www.disprot.org) includes experimentally characterized IDP and IDR sequences and the structured protein sequences in which the IDRs are embedded as well as the experimentally determined IDP and IDR functions [19–21]. This database, known as DisProt, as of this writing contains 1600 proteins, 3700 regions, and 190,100 residues, which have an overall disorder content of 21.2% [22].

Three additional databases, based on predictions of structure and disorder (discussed below), provide greatly expanded lists of likely IDPs, IDRs and their probable functions. These three databases are as follows:

1. The Mobi Database (http://protein.bio.unipd.it/mobi2/ [23]),
2. The Database of Disordered Protein Prediction (D^2^P^2^, http://d2p2.pro [24]), and
3. The DescribeProt Database (http://biomine.cs.vcu.edu/servers/DESCRIBEPROT/ [25]).

A fourth database, called the Eukaryotic Linear Motif Resource (ELM, http://elm.eu.org [26]), is a manually curated collection of short sequence motifs notable for their binding to structured protein partners. The ELM database currently contains 3527 validated ELM instances and 145 globular ELM binding domains. These ELM segments almost always occur in IDRs or IDPs [27, 28].

Amino acid compositions of IDPs and IDRs differ substantially from those of structured proteins [2, 29, 30], which enables the development of sequence-based predictors that partition IDPs or IDRs and structured proteins or regions into separate groups [31–35]. Application of these algorithms to various proteomes indicate that eukaryotes have much more disorder than prokaryotes. In one such study, the proteomes of a collection of archaea and eubacteria are predicted to have about 15–30% of their encoded residues in IDPs plus IDRs, while a collection of eukaryotic proteomes are predicted to contain 30–50% of their encoded residues in IDPs plus IDRs [36]. A recent experiment, structure/disorder prediction algorithms were applied a set of 646 proteins with regions of structure and disorder unknown beforehand to the researchers who carried out the predictions. The top three predictors exhibited balanced accuracies on this dataset ranging from 76 to 80% [37], where balanced accuracy = [(%Correct disorder predictions) + (%Correct structure predictions)]/2.

An important advance in the field of structure and disorder prediction has been to use sequence homology to identify structured domains and a disorder predictor to identify IDRs and IDPs [38]. This combined approach with the further modification of using 9 different disorder predictors gives estimates of mammalian proteome disorder to be on the order of 35–45% of the encoded residues [24]. Because this combined approach has been applied to about 17,000 (mostly prokaryotic) proteomes and compiled into the D^2^P^2^ database mentioned above [24], in most cases researchers can simply use this D^2^P^2^ to look up the results of this analysis for their proteins or organisms of interest.

## Global analysis of protein function

To better understand the biological roles of intrinsically disordered protein regions, a disorder predictor was applied to collections of proteins having the same annotation in the Swiss Protein database [39–41]. For each set of proteins with a particular annotation, one thousand matching sets of proteins with random annotations was constructed, where “matching” means same length distribution and same number of chains for the random-annotations sets. A plot of average amount of disorder in the various proteins in the one thousand matching sets gives a broad distribution, whereas each annotation-specific set gives a much narrower distribution, so the Z-score for each annotation-specific set can be estimated, where the Z-score is given as follows:

$${\text{Zi}} - {\text{score }} = \, \left( {{\text{xi }} - \, < {\text{x}} > } \right)/\sigma$$

where xi is the average predicted disorder for annotation-specific set-i, < x > is the average of the data for the one thousand matching sets, and σ is the standard deviation of the data in the one thousand matching sets.

The matching set distributions were all centered on zero, with positive scores indicating greater than average predicted disorder and with negative scores indicating greater than average predicted structure. This analysis was applied 710 Swiss Protein annotation-specific sets. Of the annotation specific sets, 238 are associated with Z-scores >  + 1 (increased amounts of predicted disorder), 170 are associated with Z-scores between + 1 and – 1 (close to the average amounts of predicted structure) and disorder), and 302 are associated with Z-scores less than – 1 (increased amounts of structure). All of these data are presented in three consecutive papers [39–41].

The 10 most structure-associated annotation specific sets (Table 1) and the 10 most disorder-associated annotation-specific sets (Table 2) from [41] have such large Z-scores that the proteins in these sets are predicted to be almost completely structured (Table 1) or disordered (Table 2).

**Table 1:** Top 10 biological processes most strongly associated with structured proteins

Keywords	Proteins (number)	Families (number)	Length (average)	Z-score
GMP biosynthesis	225	3	473	– 17.6
Amino-acid biosynthesis	7098	212	361	– 17.1
Transport	19,888	2199	378	– 14.9
Electron transport	4633	346	272	– 13.7
Lipid A biosynthesis	533	13	291	– 13.2
Aromatic catabolism	320	105	300	– 12.4
Glycolysis	2265	50	390	– 12.1
Purine biosynthesis	1208	28	445	– 11.9
Pyrimidine biosynthesis	1310	27	383	– 11.7
Carbohydrate metabolism	1797	109	404	– 11.7

**Table 2:** Top 10 biological processes most strongly associated with disordered proteins

Keywords	Proteins (number)	Families (number)	Length (average)	Z-score
Differentiation	1406	422	439	+ 18.8
Transcription	11,223	1653	442	+ 14.6
Transcription regulation	9758	1554	413	+ 14.3
Spermatogenesis	332	189	280	+ 13.9
DNA condensation	317	130	300	+ 13.3
Cell cycle	4278	612	494	+ 12.2
mRNA processing	1575	249	516	+ 10.9
mRNA splicing	716	180	459	+ 10.1
Mitosis	718	215	620	+ 9.4
Apoptosis	810	211	425	+ 9.4

The functional processes in Table 1 for mostly structured proteins are associated with enzymes (Table 1, example numbers 1, 2, 5–10) or with integral membrane proteins (Table 1, example numbers 3 and 4). Both enzymes and integral membrane proteins are almost entirely structured, although some enzymes [42] and membrane proteins [43–47] use IDRs to modulate or contribute to their respective functions. Another important category of structured proteins, but one which falls outside the top 10 list, is the set of structured signaling domains such as PDZ, SH1, SH2, etc.

In contrast to the functional processes of the structured proteins in Table 1, the functional processes in Table 2 for mostly disordered proteins heavily involve signaling and regulation. Consider one example, Table 2 example number 1, differentiation. Cellular differentiation in multicellular eukaryotes depends upon sets of gene regulatory pathways as well as upon the downstream pathways that follow from the expression of certain genes at certain times and locations, leading to the promotion of new cell types. The gene regulators themselves, that is the transcription factors, are highly disordered [48, 49], and this disorder depends on both post-transitional modification and INDELs arising from alternative splicing to increase signaling complexity [50]. Furthermore, several differentiation-associated transcription factor families exhibit a strong correlation between increasing fractions of predicted disorder and increasing organism complexity as estimated by numbers of different cell types [51], suggesting that increased organism complexity requires increased transcription factor complexity to handle the increasing complexity of the gene regulation. Also, the expressed proteins resulting from the gene regulation and underlying cellular differentiation of both plants and animals show a high occurrence of predicted disorder [52–57].

All these and many other observations suggest that the classic sequence → structure → function model is clearly an oversimplification, and in reality, a relation between protein sequence and function can be viewed as a structure–function continuum concept, in which the actual protein structure–function relationship is described by the more convoluted ‘one-gene—many-proteins—many-functions’ model [58, 59]. This is because proteins are characterized by a very complex and heterogeneous spatiotemporal structural organization, possessing foldons (independent foldable units of a protein), inducible foldons (disordered regions that can fold at least in part due to the interaction with binding partners), non-foldons (non-foldable protein regions), semi-foldons (regions that are always in a semi-folded form), and unfoldons (ordered regions that have to undergo an order-to-disorder transition to become functional) [60–63]. This intricate structural, mosaic-like ‘anatomy’ of proteins defines their unique molecular ‘physiology’, where differently (dis)ordered structural elements might have well-defined and specific functions [59], thereby allowing a protein molecule to be multifunctional and to interact with, to be regulated by, and/or to regulate multiple structurally unrelated partners.

Given the lack of sufficient coverage of IDPs and IDRs in current biochemistry and cell biology curricula, we suspect that many developmental biologists are studying cell communication and signaling without realizing the important underlying contributions being made by IDPs and/or IDRs in the very proteins they are investigating. We hope that this brief introduction to IDPs and IDRs and this collection of papers focused on this topic will raise awareness of these proteins in the cell communication and signaling community.

## The IDP/IDR in signaling collection

Our collection consists of thirteen papers, which are very briefly described as follows:

1. Cell signaling pathways cannot be fully described without understanding how intrinsically disordered protein regions contribute to its function. Bondos et al. [64] opens this collection by providing an overview of the breadth of roles of IDPs and IDRs in cell signaling, and the attributes that intrinsic disorder can provide a cell signaling pathway, including the ability to amplify, regulate, or tune the signal, and the ability of integrate multiple signals into a single response. This review also highlights the critical role of intrinsically disordered proteins for signaling in widely diverse organisms (animals, plants, bacteria, fungi), in response to a wide array of chemical and physical signaling, in every category of cell signaling pathways (juxtacrine, and paracrine) and at each stage (ligand, receptor, transducer, effector) in the cell signaling process. The universal presence of intrinsic disorder in different stages of diverse cell signaling pathways suggest that more mechanisms by which disorder functions remain to be discovered.
2. Liu [65] analyzes consequences of codon usage bias associated with the genetic code generation, where most amino acids are encoded by two to six synonymous codons. Codon usage bias describes the organism-specific preference for certain synonymous codons and represents a common feature universally found in all genomes examined. Although, for a long time, it was believed that the synonymous codon mutations are silent, this article provides a comprehensive analysis of the recent literature to make a strong opposing case. Accumulated evidence unequivocally shows that synonymous codon mutations are not silent at all, and, instead, codon usage has multiple functional roles. In fact, codon usage plays a role in regulation of the translation kinetics and co-translational protein folding and shows significant effects on protein structure, gene expression, and translation efficiency and accuracy, with disordered regions showing greater sensitivity to such synonymous mutations as compared to structured regions.
3. Pelham et al. [66] review the key proteins regulating circadian rhythms in three model organisms, mice, neurospora, and drosophila, showing that all are highly enriched in protein disorder, and that disorder is pervasively conserved amongst the circadian clock proteins in the crown eukaryotes (i.e., lineages descending from LECA, Last Eukarytotic Common Ancestor). Clock proteins utilize intrinsic disorder for post translational modifications, protein–protein interactions, and complex regulation, thereby indicating that conserved intrinsic disorder is essential for optimal circadian timing.
4. Parico and Partch [57] continue discussion of the roles of intrinsic disorder in controling and regulation of circadian rhythms by describing the involvement of intrinsically disordered C-terminal tail in functionality of the cryptochromes (CRYs), which are blue light sensitive flavoproteins involved in the circadian rhythms and magnetic field sensing. CRY contains two functional domains, ordered N-terminal photolyase homology region (PHR) and intrinsically disordered C-terminal domain. The authors discuss a general and evolutionarily conserved model for CRY function, where PHR is necessary and sufficient to generate circadian rhythms serving as a platform for binding of other components of the circadian clock network, whereas intrinsically disordered C-tail modulates the amplitude and periodicity of circadian rhythms undergoing reversible interactions with various protein partners.
5. Creamer [67] reviews a long disordered region at the C-terminus of the catalytic subunit of the serine/threonine phosphatase calcineurin. This protein acts as a crucial connection between calcium signaling and the phosphorylation states of various substrates. It contains an autoinhibitory domain and a Ca^2+^/calmodulin binding site that together provide an on–off switch for regulating calcineurin’s phosphatase activity, which in turn plays key roles in many different phosphorylation-regulated signaling pathways.
6. Seiffert et al. [68] point out that, like essentially every other eukaryotic single pass membrane protein, the Class 1 cytokine receptors (C1CRs) contain long intrinsically disordered intracellular domains (ICDs), which are used to orchestrate key biological processes, such as differentiation, immunity, growth, and proliferation. ICDs of C1CRs contain numerous short linear motifs (SLiMs), which are used for transient interactions with multiple signaling partners. The fact that many SLiMs are overlapping emphasizes the involvement of these disorder-based functional features in a complex regulation of functional interactions, including network rewiring by isoforms. The authors conclude that C1CR-ICDs carry both organizational and operational features and are intensively used in orchestration of complex cellular signaling processes.
7. Skalidi et al. [69] dedicated their review to the systematic analysis of the roles of intrinsically disordered regions (IDRs) in three gigantic, multi-enzyme complexes, pyruvate dehydrogenase, oxogluterate dehydrogenase, and fatty acid synthase, known as “metabolons”. These complexes regulate the synthesis of their products—acetyl-CoA, α-ketoglutarate, and palmitic acid, respectively, with conserved disordered regions within metabolons determining the yield of these metabolites. Furthermore, this IDRs tend to be regulated by intricate phosphorylation patterns, act as act as spatial constraints confining enzyme communication, and tether functional protein domains. Importantly, metabolites synthesized by these metabolons have a broad spectrum of non-metabolic signaling functions and play important roles in intracellular communication, inflammation, and malignant transformation.
8. Hesgrove and Boothby [70] dedicate their review to the analysis of the available data on the roles of intrinsically disordered proteins in extreme stress tolerance of tardigrades (water bears or moss piglets), microscopic animals famous for their capability to survive a broad range of environmental extremes that would kill almost any other animal. In fact, these eight-legged segmented micro-animals have a reputation as the toughest animals on the planet that can tolerate 1000 times more radiation than other animals, withstand extreme temperatures [from − 272 °C (− 458 °F; 1 K) to 151 °C (304 °F)] or pressure (from the extremely low pressure of a vacuum to more than 1200 times atmospheric pressure), and can survive momentary shock pressures up to about 1.14 gigapascals (an equivalent of direct bullet impact) or complete dessication. The authors provides data showing that tardigrade cytoplasmic-, secreted-, and mitochondrial-abundant heat stable intrinsically disordered proteins (collectively termed Tardigrade Disordered Proteins, TDPs) confer stability in the face of variety of extreme environmental conditions including an extraordinary degree of desiccation. It is also emphasized that these protective TDPs act by yet-to-be determined molecular mechanisms, as comprehensive and holistic understanding of the fundamental mechanisms of their functions and a detailed knowledge of their properties defining the capability of TDPs to function via those mechanisms are still missing.
9. Kragelund et al. [71] provide a general discussion of the roles of the αα-hubs, which are small α-helical domains found in large, modular proteins that bind and regulate intrinsically disordered transcriptional regulators. Then, using a set of comparative structural biology tools, they discover the new member of the αα-hub group, harmonin-homology-domain (HHD, also named the harmonin N-terminal domain, NTD), which is found in several proteins: cerebral cavernous malformation 2, harmonin, regulator of telomere elongation 1, and whirlin. This new member of the αα-hubs not only expands functionality ascribed to this group of hub domains, but also provides an example of how the discoveries in one member may reveal discoveries in others. The αα-hubs may serve as unique models for generating signal specificity and fidelity. These concepts advance our understanding of the complex functionality of hub proteins and the roles of IDRs in controlling signaling networks.
10. In their research article, Shao et al. [72] used a set of biochemical, bioinformatics, and biophysical methods to characterize a small chloroplast protein, CP12, from the marine diatom *Thalassiosira pseudonana*. For a long time, CP12, which is conserved in many diatoms and has a number of important functions in various photosynthetic organisms, playing a role in the redox signaling pathway involved in the regulation of the Calvin Benson Bassham (CBB) cycle, has been overlooked. The authors show that CP12 is constitutively expressed in all growth phases of dark-treated and in continuously light-treated *T. pseudonana* cells. This protein was shown to have coiled coil and disordered regions and can have one-to-many functions beyond the dark downregulation of CBB enzymes, serving as possible signaling proteins coordinating multiple cell actions in response to fluctuating environments.
11. Kolonko et al. [73] investigate a member of the family of bHLH-PAS transcription factors, *Drosophila melanogaster* germ-cell expressed protein (GCE), which is a paralog of the juvenile hormone (JH) receptor, methoprene-tolerant protein (MET). Although both GCE and MET proteins act as JH receptors and prevent precocious differentiation during *D. melanogaster* development, their functions are tissue specific and not redundant. In addition to the conserved bHLH and PAS domains, these proteins contain long dissimilar C-terminal fragments (GCEC, METC). The authors show that GCEC behaves as a coil-like intrinsically disordered protein, being less compact than METC and containing more disorder-based binding motifs, molecular recognition feature (MoRFs). GETC is capable of interaction with the ligand binding domain (LBD) of the nuclear receptor Fushi Tarazu factor-1 (FTZ-F1) and at least partially folds as a result of complex formation. It is likely that the aforementioned dissimilarity of GCE and MET functions and their tissue-specific differences arise from their long disordered GCEC and METC regions that have distinctive sequences, shapes, and functions.
12. Peifer et al. [74] describes roles of the long IDR in functionality and stability of the non-receptor tyrosine kinase Abelson (Abl). Since Abl is a crucial player in oncogenesis, inhibitors targeting this kinase serve as prototypes of targeted therapy. In addition to be a proto-oncogene, Abl is implicated in cell differentiation, cell division, cell adhesion, and stress response, acts as critical regulator of normal development, and play conserved roles in regulating cell behavior, brain development, and morphogenesis. Because only one *Abl* gene is present in *D. melanogaster*, flies serves as great model for the functional analysis of this multi-domain scaffolding protein. In Drosophila, Abl protein is 1,620-residue-long protein containing intrinsically disordered N-terminal region, followed by SH3, SH2, Abl kinase domains linked via a long IDR to the C-terminally located F-actin binding domains. The authors investigated roles of this long intrinsically disordered linker (~ 900 residues) connecting kinase and F-actin binding domains in Drosophila Abl. Based on the analysis of the Abl∆IDR variant, where the entire IDR was deleted, it was concluded that this IDR is essential for all aspects of protein function during embryogenesis, embryonic and adult viability, as well as for cell shape changes. Furthermore, it can regulate cytoskeleton during embryonic morphogenesis, and plays an important role in regulation of protein stability.
13. Recent years witnessed dramatic increase in the interest of researchers to membrane-less organelles or biomolecular condensates that represent the non-stoichiometric assemblies of biomolecules defined by spatial concentration of cellular components, are formed through the process of phase separation, and commonly involve proteins containing IDRs or ordered oligomerization domains capable of multivalent interactions and thereby drive higher-order assembly. The roles of promiscuous IDRs and oligomerization domains in biogenesis of biomolecular condensates are poorly understood. To fill this gap, Emenecker et al. [75] combined quantitative microscopy with numbers and brightness analysis to investigate the aging, material properties, and oligomeric state of the biomolecular condensates in vivo. As a model, the authors used cytoplasmic condensates formed by a transcription factor integral to the auxin signaling pathway in plants, auxin response factor 19 (ARF19). ARF19 contains a large central glutamine-rich IDR and an ordered C-terminal Phox Bem1 (PB1) oligomerization domain. This analysis revealed that the morphology and material properties of ARF19 condensates can be modulated by the IDR amino acid composition, which, however, did not have noticeable impact on the distribution of oligomeric species within condensates.

## Call for papers

It is clear that articles assembled into this special issue only scratched the tip of the iceberg and many important questions related to the role of intrinsic disorder in regulation of cell signaling and communication are waiting to be asked and answered. Cell Communication and Signaling encourages additional submissions on this research topic. If you believe that you can add to one or more questions related to this subject, please to submit your manuscript to become a part of this CCAS thematic series.
